# “Welcome to the World of the Plastic Beach”

**DOI:** 10.3201/eid2104.AC2104

**Published:** 2015-04

**Authors:** Byron Breedlove

**Affiliations:** Centers for Disease Control and Prevention, Atlanta, Georgia, USA

**Keywords:** art science connection, emerging infectious diseases, emerging viruses, environment, gyres, invasive species, plastic, art and medicine, welcome to the world of the plastic beach, Pam Longobardi, ghosts of consumption/archaeology of culture (for Piet M.), about the cover

**Figure:**
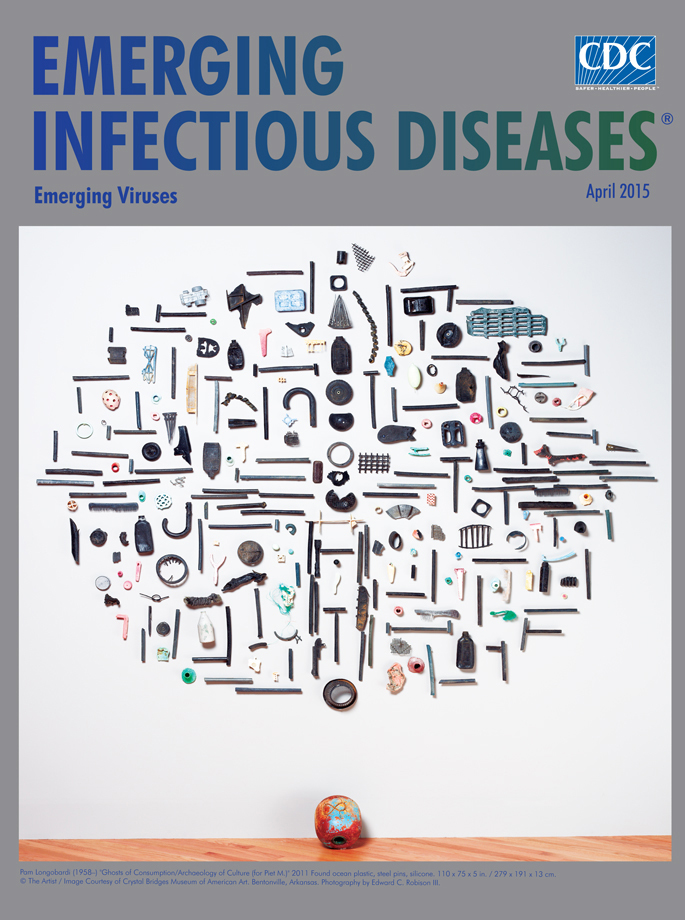
**Pam Longobardi (1958–) Ghosts of Consumption/Archaeology of Culture (for Piet M.) (2011) Found ocean plastic, steel pins, silicone (110 × 75 × 5 in/279 × 191 × 13 cm)** © The Artist/Image Courtesy of Crystal Bridges Museum of American Art.

“Welcome to the World of the Plastic Beach” is the title and refrain of the second song from the 2010 concept album *Plastic Beach* by the British virtual band Gorillaz. Damon Albarn, who writes the songs, was moved to explore themes of disposable commodities when he noticed all the plastic mixed with the sand near his beach house.

That was four years after Pam Longobardi, an Atlanta-based artist and professor of art at Georgia State University, had encountered “mountainous piles of plastic the ocean regurgitated on remote Hawaiian beaches.” Longobardi describes herself as a conceptual artist focused on exploring “the cultural archaeology of our time.” This month’s cover image, *Ghosts of Consumption/Archaeology of Culture (for Piet M.)*, is one of the most recognized works in her award-winning, ongoing Drifter’s Project. It documents the impact of plastic marine debris as it journeys around the world’s oceans, coalescing inside giant oceanic currents known as gyres and concentrating the plastic into areas such as the Great Pacific Garbage Patch that floats north of Hawaii.

Hundreds of plastic objects—taken from the tens of thousands of pounds of plastic debris Longobardi has removed from beaches—are on display in *Ghosts of Consumption*. Perched on steel pins, this assemblage of flotsam suggests an archeologic or forensic exhibition and invites the viewer to step forward and scrutinize each item. Displayed among this ocean-worn debris are household items, kitchen utensils, toys, and sporting goods; interspersed among them are the numerous black tubes that provide unity and rhythm to the installation. Many of these items are not recognizable; their original shapes have been distorted, smoothed, and rounded by the oceans’ crucible of tides, currents, heat, friction, and chemistry.

*Ghosts of Consumption*, dedicated to the Dutch artist Piet Mondrian, recalls Mondrian’s 1915 painting *Pier and Ocean*. Celebrated for its precise visual arrangement of intersecting horizontal and vertical lines, *Pier and Ocean* captures the pulse and rhythm of the ocean. The horizontal lines symbolize the ocean’s surface, rendering its transient troughs and ridges uniformly, and the vertical lines denote the man-made pier that juts into the ocean, its form defined by the intersecting planes.

Longobardi’s montage, representing the collision of nature and consumerism, also comprises an oval grid of horizontal and vertical lines, shapes, and forms. The intersection and contrast of the black tubes and distorted artifacts of collected rubbish disrupt the work’s balance and harmony. According to the artist, the horizontal elements symbolize the natural world, and the vertical grid represents the human element, in particular, the consumer-based cultures of the world. “I created this work as an homage to Mondrian, an artist whom I admire. Mondrian’s work was his response to the relationship between humans and the ocean in 1914, and mine is a response to our relationship to the ocean 100 years later.”^1^

In *Ghosts of Consumption*, Longobardi uses art to engage our minds and our hearts in an unavoidable conversation about the consequences of disposability to the ocean environment. Longobardi describes her installations as “being preferentially in a transitive state, such that they may be reabsorbed into culture, commerce, or industry, as the technology develops to return plastic into oil.”

Exactly how much plastic is in the ocean cannot be precisely measured. Plastics are estimated to comprise 60%–80% of all marine litter, perhaps 90%–95% in some areas. More than an eyesore, this debris harms marine biota and allows invasive species to hitchhike around the globe. The United Nations is among those who have flagged this problem, noting that “Communities of microbes have been discovered thriving on microplastics at multiple locations in the North Atlantic. This ‘plastisphere’ can facilitate the transport of harmful microbes, pathogens and algal species.”

*Ghosts of Consumption* focuses on the global consequences of disposability. The late Lewis Thomas wrote about the interdependence of life on earth and suggested the earth itself of being “most like a cell.” This perspective offers another way to view Longobardi’s installation, which resembles the contour of a cell, its myriad black tubes analogous to the microtubules within a cell’s cytoskeleton. Once pristine and healthy (more like that Mondrian painting), the cell is now infested with various rods, filaments, and spheres of plastic debris and flotsam, items that emerged from the ocean reshaped and mutated from their original forms.

The ideas that fueled Longobardi’s creation of *Ghosts of Consumption* appear in many of the articles in this issue and should also resonate with readers and researchers. Environmental degradation and encroachment, global travel and commerce, and climate are factors that provide opportunities for viruses to emerge in novel and expanded niches and to infect new host populations. Such human-derived factors allow these emerging pathogens greater dispersal and more opportunities to spill over to humans or other hosts.
